# Innate immune biology in age-related macular degeneration

**DOI:** 10.3389/fcell.2023.1118524

**Published:** 2023-02-28

**Authors:** Karina Ascunce, Rahul M. Dhodapkar, Deven Huang, Brian P. Hafler

**Affiliations:** ^1^ Interdepartmental Neuroscience Program, Yale University, New Haven, CT, United States; ^2^ Department of Ophthalmology, Roski Eye Institute, University of Southern California, Los Angeles, California; ^3^ Choate Rosemary Hall, Wallingford, CT, United States; ^4^ Department of Ophthalmology and Visual Science, Yale University, New Haven, CT, United States; ^5^ Department of Pathology, Yale University, New Haven, CT, United States

**Keywords:** neuroinflammation, age-related macular degeneration (AMD), neurodegeneration, neuroimmune biology, innate immunity

## Abstract

Age-related macular degeneration (AMD) is a neurodegenerative disease and a leading cause of irreversible vision loss in the developed world. While not classically described as an inflammatory disease, a growing body of evidence has implicated several components of the innate immune system in the pathophysiology of age-related macular degeneration. In particular, complement activation, microglial involvement, and blood-retinal-barrier disruption have been shown to play key roles in disease progression, and subsequent vision loss. This review discusses the role of the innate immune system in age-related macular degeneration as well as recent developments in single-cell transcriptomics that help advance the understanding and treatment of age-related macular degeneration. We also explore the several potential therapeutic targets for age-related macular degeneration in the context of innate immune activation.

## Introduction

Age-related macular degeneration (AMD) is a neurodegenerative disorder characterized by the presence of drusen, extracellular deposits, followed by retinal pigment epithelium (RPE) and photoreceptor loss. AMD is rarely seen before age 55, after which prevalence increases with increasing age (Klein et al., 2010). Several classification systems exist for AMD, with disease taxonomy defined based on the frequency and size of macular drusen, the presence of pigmentary abnormalities, and the presence of geographic atrophy or neovascularization (Fleckenstein et al., 2021). AMD is commonly subcategorized into neovascular (wet) disease where choroidal vascular changes are present, and non-neovascular (dry) disease where such changes are absent. While only a minority (10%–15%) of patients will develop neovascular disease, wet AMD is responsible for a majority of cases of sudden vision loss in the disease (Jager et al., 2008).

AMD is a multifactorial disease, with a complex set of risk factors, like age, environment, and genetic susceptibility; the heritability of late stage AMD has been estimated to be up to 71%, higher than most complex age-related diseases (Fleckenstein et al., 2021). The strongest risk factor for AMD is age. Aging is a critical component of AMD pathology; during aging, injury, or inflammation, resident microglia migrate to the affected site. Microglial infiltration can trigger a response involving the infiltration of other myeloid-derived cells, including peripheral monocytes and monocyte-derived macrophages. CCL2 and CX2CL1 chemokines, for instance, are implicated in AMD-related subretinal microglial and macrophage infiltration (Raoul et al., 2010). Expression patterns of cytokine and cytokine receptors change as biological age and state progress, indicating these inflammatory mechanisms may be involved in AMD development.

Modifiable risk factors for AMD include smoking, diet, and physical activity. Smoking has been shown to increase the chance of developing AMD by two to four times, and cessation reduces AMD risk (Velilla et al., 2013). Consumption of foods with lutein, zeaxanthin, fish oil, and polyunsaturated fatty acids have been shown to decrease the chance of late stage AMD. These insights have led to the formulation of the AREDS vitamin supplementation regimen, a mainstay of therapy for non-neovascular AMD (AREDS2 Research Group et al., 2012).

While the precise pathogenesis of AMD remains unknown, several lines of evidence implicate drusen, the hallmark lesions of AMD. Several factors, including amyloid, complement, and 7-ketocholesterol, have been isolated from drusen and are hypothesized to contribute to AMD progression (Mullins et al., 2000; Rodriguez et al., 2014). The development of neovascular AMD in particular is strongly associated with the vascular endothelial growth factor (VEGF) family of cytokines and resultant signaling through Flt-1 (Nork et al., 2011). The identification of this link has been key to the development of a host of anti-VEGF therapies that have been used in the treatment of neovascular AMD (Solomon et al., 2019).

In the retina, glial cells support photoreceptor function by providing physical and metabolic support through a number of mechanisms such as transporting nutrients and clearing debris. The three broad types of glial cells in the retina are microglia, astrocytes and Müller cells (Reichenbach and Bringmann, 2019). Retinal microglia respond to retinal injury and mediating neuroinflammation, where retinal macroglia–astrocytes and Müller cells–maintain retinal homeostasis through regulation and transport of ions, glucose, and neurotransmitters. Microglia and macroglia involvement have both been implicated in AMD pathogenesis.

Methods exploring the underpinnings of AMD are rapidly developing. In recent years, scientists have been using single-cell RNA sequencing (scRNA-seq) to analyze the pathogenesis and etiology of diseases whose origins remain unclear. Due to the genetic complexity of AMD, it has been difficult to precisely identify the cell-types associated with AMD development. In 2019, researchers used scRNA-seq to develop the first single-cell transcriptomic atlas of the human retina, a full record of the transcriptomes of each cell type (Menon et al., 2019). Use of the transcriptomic atlas predicts Müller glia and astrocytes have a role in the pathogenesis of AMD (Menon et al., 2019).

A 2021 study built upon these findings by focusing on the genes transcribed instead of the cells. This study used scRNA-seq to obtain the transcriptomes of 93,000 retinal cells; in addition to using the data to transcriptionally identify differences in retinal neurons, researchers also assessed the cell-type expression levels of each AMD risk gene (Lyu et al., 2021). Through this method, 23 risk genes were found to be cell-type-specific genes. For instance, CFH, a gene strongly associated with AMD, was determined to be specifically expressed in endothelium cells. This relationship implied that endothelial cells may play a role in the development of AMD. In addition, C3 and CFI, two other AMD genes, were found to be preferentially expressed; C3 by microglia and astrocytes, and CFI by astrocytes, endothelium, and Müller cells. Although the complete pathogenesis of AMD remains unclear, scRNA-seq allows scientists to better understand which cell types are involved in the disease’s development.

A growing body of research has shown that AMD pathogenesis is associated with neuroimmune interactions; most recently, neovascular AMD pathology was associated with COVID-19 infection severity and morbidity, providing just one example of the potential neuroimmune interactions involved in AMD (Yang et al., 2022). The blood-retina barrier protects the retina, making it an immune-privileged part of the eye; still, innate immune interactions may influence the progression of this neurodegenerative disorder. Immune privilege in the eye is characterized in part by Müller glia and astrocytes–in addition to endothelial cells, pericytes, and perivascular macrophages, among others–modulating the trafficking of cells in and out of the retina. However, the innate immune response–including macrophages, monocytes, neutrophils, and natural killer T cells–may serve to play a role in neurodegenerative diseases, including AMD (Novellino et al., 2020; Dhodapkar et al., 2022). Innate immune cells contribute to the maintenance of the blood-retina barrier, vascularization, and inflammation, and their disruption or dysfunction may have implications for the development of AMD. This article focuses on the role of these immune cells in AMD pathogenesis.

### Macrophages

Macrophages are a diverse group of cells that serve several functions across tissue repair, immunity, and homeostasis (Wynn et al., 2013). In the central nervous system, macrophages can secrete proinflammatory factors; while microglia constitute the bulk of resident myeloid cells in the central nervous system, monocyte-derived macrophages may become activated depending on the surrounding milieu (Yang et al., 2020). Macrophages along the outer collagenous zones of Bruch’s membrane have been shown to be present in AMD lesions (Killingsworth et al., 1990). The phagocytic ability of cultured human RPE has been found to be decreased in AMD donors (Inana et al., 2018). Macrophages have been shown to become activated in several different ways. A widely used system classifies macrophage activation as either M1 (classic activation) or M2 (alternative activation); however, recently more activation states have been identified. M1 macrophages, specifically, are known to activate phagocytic and antitumor inflammatory responses (Liu et al., 2021). In neovascular AMD monocytes and in the neovascularization-choroidal rodent model, M1 macrophages had a proangiogenic effect; in both models, angiogenesis was mediated by TNF-α, whose inhibition led to a decrease of choroidal neovascularization (Hagbi-Levi et al., 2021). Perivascular macrophages, cells distinct from microglia, are present in the retina and are believed to contribute to the blood-retinal barrier maintenance during homeostasis. During photoreceptor degeneration, these cells may be recruited to sites of damage (Mendes-Jorge et al., 2009). Although the role of these peripheral macrophages in the perivascular choroidal space in AMD is not yet fully understood, alterations to their function may be associated with age-related changes in the choroidal vasculature (Yang et al., 2020).

In the murine model, macrophage ablation by CSF1R blockade leads to choroid vascular atrophy in addition to structural and angiogenic RPE dysfunction, suggesting that macrophages residing in the choroid may serve an important role in structural maintenance of the RPE and choroid vasculature (Yang et al., 2020). One signaling pathway that helps maintain retinal immune homeostasis is the interferon-beta signaling pathway, which in the murine model has been used to reduce microgliosis, macrophage response, and choroidal neovascularization (Lückoff et al., 2016). In cases of photoreceptor degeneration, monocyte infiltration can precede microglia infiltration during the early stages of the immune response (Karlen et al., 2018). This finding suggests that monocyte-derived macrophages along with microglia may play a role in the immune response in AMD (Karlen et al., 2018). In retinitis pigmentosa (Tebbe et al., 2020) monocyte-derived macrophages may contribute to cone death as their inhibition protects against cone degeneration in the rd10 mouse model of retinitis pigmentosa (Funatsu et al., 2022). Given their overall role in choroidal vascularization and RPE maintenance, targeting these cells may be a potential therapeutic approach for AMD, particularly as it relates to limiting angiogenesis and chronic inflammation. It may be possible to explore stem cell therapeutic potential to restore their phagocytic function as genetically-modified stem cells have previously been shown to promote turnover of perivascular macrophages in the macaque brain (Soulas et al., 2009).

### Monocytes

Monocytes, as part of the immune system, can differentiate into macrophages or dendritic cells. Monocyte-derived macrophages, as aforementioned, appear to play a role in AMD, but the primary difference between these two cells is their localization; monocytes circulate in the bloodstream where macrophages infiltrate tissue. Peripheral monocyte counts, in particular, have been shown to be higher in AMD patients versus controls (Xue et al., 2021). In intermediate and advanced AMD, monocyte phagocytic function is shown to be reduced about 40% across several monocyte subtypes compared to controls (Gu et al., 2021). Gu et al. found that this effect could be ameliorated by application of glatiramer acetate, which may provide a therapeutic avenue for AMD (Gu et al., 2021). Glatiramer acetate is an immunomodulatory polypeptide that is used in the treatment of relapsing or remitting multiple sclerosis (Gran et al., 2000). Increased TNF-α is also associated with monocyte activation, as patients with higher prevalence of choroidal neovascularization demonstrated high levels of monocyte TNF-α (Cousins et al., 2004).

In atrophic, or dry, AMD, CCR2+ monocytes have been identified in the subretinal space (Sennlaub et al., 2013). In the mouse model, these monocytes were also shown to possibly contribute to photoreceptor degeneration. Furthermore, CD163+ monocytes and macrophages may have a role in AMD pathology and progression (Swayze et al., 2022). In late stages of AMD, these changes in monocyte function can potentially be modified by the P2X7 receptor, which is expressed in a variety of cells, especially in monocytes, and can function as a scavenger receptor mediating phagocytosis (Drysdale et al., 2022). The P2X7 receptor mediated membrane fluidity, which was reduced across several types of leukocytes, including monocytes, in advanced AMD. In mice, impaired cholesterol clearance by monocytes in the murine model triggers several events leading to the development of cholesterol-rich drusen deposits underneath the RPE, a hallmark of AMD (Ban et al., 2018). These drusen deposits may be associated with disease progression and may provide a potential therapeutic target for AMD.

### Natural killer cells

Natural killer (NK) cells are a component of the innate immune system, affecting cytokine production and cytotoxicity, with antiviral and antitumoral effects by killing cells that downregulate the MHC Class I self-antigen presentation system (Vivier et al., 2008; Bern et al., 2019). In a genotyping study of AMD and control patients, the NK receptor AA haplotype was, in combination with the human leukocyte antigen (HLA)-Cw*0701 allele, associated with AMD (Goverdhan et al., 2008). In another study of gene expression profiles in AMD, there was a lower prevalence of resting NK cells in AMD versus control, but the C1S, ADM, and 1ER5L genes were shown to have a positive correlation with activation of NK cells, among others, along AMD progression (Zeng et al., 2021). Further experiments in mice models have shown that *in vivo* depletion of interferon-γ-secreting NK cells lead to reduced choroidal neovascularization (CNV) (Lee et al., 2014).

### Neutrophils

Neutrophils are a type of leukocyte that drive both acute and chronic inflammation (Kolaczkowska and Kubes, 2013). As with monocytes, the P2X7 receptor mediates membrane fluidity, which is reduced in neutrophils and other leukocytes in advanced AMD (Drysdale et al., 2022). Furthermore, the neutrophil-to-leukocyte ratio is higher in patients with neovascular AMD (Niazi et al., 2019). Increased INFλ levels lead to LCN-2 upregulation and neutrophil activation; inhibition of this INFλ inflammatory signal, through AKT2 inhibition, leads to decreased LCN-2-mediated neutrophil infiltration, which may provide a novel therapeutic approach to early dry AMD (Ghosh et al., 2019).

Neutrophils are characterized by their strategy for pathogen eradication, which is known as a neutrophil extracellular trap (Kolaczkowska and Kubes, 2013). Neutrophil extracellular trap formation has previously been associated with retinal diseases (Ghosh et al., 2019; Wang et al., 2018). Aβ1-40 the main amyloid-beta component of the drusen characteristic of AMD may promote neutrophil extracellular trap formation in AMD through the Toll-like receptor 4 and neutrophil NADPH oxidase pathways (Chen et al., 2022). PAD4 inhibition in the mouse model led to reduced neutrophil extracellular trap formation, providing a potential therapeutic option for AMD (Chen et al., 2022). PAD4 inhibition is currently used to treat rheumatoid arthritis and lupus, among other autoimmune diseases. This finding suggests a possible role for neutrophils in AMD that needs to be explored further.

### Dendritic, B, and T cells

Dendritic cells are innate immune cells that activate lymphocytes, in addition to capturing and processing antigens (Banchereau and Steinman, 1998). Early dendritic cell recruitment occurs during retinal injury (Lehmann et al., 2010). In a laser-induced CNV mouse model of AMD, both wild-type and dendritic-cell-deficient mice exhibited similar levels of CNV area, (Droho et al., 2021). Dendritic and B cells alike are yet to be strongly linked with AMD progression.

T cells are lymphocytes that can be activated by dendritic cells. Chemokine profiles of CD4^+^ and CD8^+^ patients differ between AMD and control conditions (Choi et al., 2022). Furthermore, in AMD versus control gene expression analysis, the AMD condition exhibited lower resting CD4^+^ memory T cells (Zeng et al., 2021). T cells may become activated in response to oxidative stress (Cruz-Guilloty et al., 2014). T-helper (Th) 1 T cells were found at lower frequency in patients with neovascular AMD (Singh et al., 2017). CD56^+^ CD28- T cells in the peripheral blood are higher in AMD versus control patients, suggesting a possible function of T cells in AMD (Faber et al., 2013).

CNV lesions in the murine model triggered T cell immune response as well, with an increase in IL-17-producing γδT-cells through C5a (Coughlin et al., 2016). In CD4^+^ T cells, C5a stimulates IL-17 production, which may imply a role for C5a in AMD and T lymphocyte activation (Liu et al., 2011). Natural killer T (NKT) cells make up a small percentage of innate immune cells. NKT cells are implicated in CNV, and in Cd1d-restricted invariant and NKT-deficient mice, there are lower levels of CNV and VEGF, indicating the potential role of NKT cells in AMD (Hijioka et al., 2008).

Possible therapeutic targets include targeting the interferon-beta signaling pathway or the AKT2 signaling pathway (Lückoff et al., 2016; Ghosh et al., 2019). Targeting proteins such as PAD4 or C5a may produce similar results. Recent clinical trials for geographic atrophy have shown success at targeting the complement system (Coughlin et al., 2016; Chen et al., 2022). Therapeutic targets implicated in other neurodegenerative diseases such as glatiramer acetate, which is primarily used in MS has shown some efficacy in reversing AMD monocyte pathology *in vitro* (Gu et al., 2021). One limitation of potential therapies is their use in the murine model, which lacks an anatomical macula (Pennesi et al., 2012).

## Conclusion

Progress in understanding the peripheral immune response in AMD has been made and future research on human retinas with AMD using single-cell approaches will further help the field. Furthermore, the development of non-human primate models of AMD or human retinal organoids derived from human pluripotent stem cells from patients with AMD may facilitate the translational development therapeutic interventions for AMD targeting immune dysregulation.
